# A rehabilitative approach beyond the acute stroke event: a scoping review about functional recovery perspectives in the chronic hemiplegic patient

**DOI:** 10.3389/fneur.2023.1234205

**Published:** 2023-09-15

**Authors:** Teresa Paolucci, Francesco Agostini, Elena Mussomeli, Sara Cazzolla, Marco Conti, Francescapia Sarno, Andrea Bernetti, Marco Paoloni, Massimiliano Mangone

**Affiliations:** ^1^Department of Medical, Oral and Biotechnological Science (DSMOB), University “G. d’Annunzio” of Chieti-Pescara, Chieti, Italy; ^2^Department of Anatomy, Histology Forensic Medicine and Orthopedics, Sapienza University, Rome, Italy

**Keywords:** chronic stroke, motor impairment, stroke sequelae, rehabilitation, hemiplegic patient, recovery

## Abstract

**Background:**

Stroke is a main cause of disability worldwide and its neuro-rehabilitative management is not limited to the acute phase but requires continuity in the rehabilitation approach especially in the chronic phase. The aim of this scoping review was to highlight the different treatment opportunities available in neurorehabilitation, effective for patients with chronic stroke sequelae, not only in terms of maintaining motor function but also improving it.

**Methods:**

The literature search was conducted using the following databases: MEDLINE (PubMed), PEDro, Scopus, Web of Science (WOS), Cochrane from 2012 to February 2023. We selected Randomized Clinical Trials in English dealing with neurorehabilitation strategies in chronic hemiplegic patients after stroke focusing on motor function, muscular strength, gait, postural balance, spasticity, and quality of life.

**Results:**

According to the inclusion criteria, 47 articles were selected for our review. All of them were analyzed following the primary outcome and the rehabilitation technique used. Despite the different protocols used within the same technique and despite the chronicity of the disease, all studies report an improvement after the rehabilitation treatment of motor function and quality of life.

**Conclusion:**

The literature analyzed invites us to reflect respect to neurorehabilitation approach to the patient with chronic stroke sequelae often considered to have as its objective the maintenance of the present motor function and contain disability: instead, the review reports how, even in chronicity, the patient always reports margins of statistically and clinically significant improvement. The chronic stroke rehabilitation over 6 months has been proved effective in obtaining recovery in different settings.

## 1. Introduction

Stroke is one of the leading causes of death worldwide (1, 2) and it often leads to severe neurologically based disability in adults (3). Its consequences can affect cognitive, psychological, social and physical integrity: the assessment should consider the patient comprehensively in order to allow the best possible return to everyday life. Motor impairment is the most frequently recorded disability after a stroke episode (4), for this reason patient’s rehabilitation is essential to achieve a good recovery (5). In agreement with the literature, chronic stroke phase begins 6 months after the acute event: it is considered that the best recovery plateau is reached at this point, in fact there is much evidence in favor of the improvements achieved in the acute and subacute phases. It is estimated that 80% of stroke patients achieve their maximum recovery within the first 3 months, reaching 95 and 100%, respectively, after the first semester and 1 year (6, 7). One of the causes might be the tendency to give more importance to the reacquisition of walking than to the fine motor skills, for instance it is estimated that upper limb motor impairment persists in 55–75% of chronic stroke patients (8, 9). However, stroke patients often need a longer period of rehabilitation, after the first phase that is provided in a hospitalization context, whereas the patient needs to experiment and integrate the motor and cognitive skills re-acquired after the stroke in his/her social context, both family and work. As a matter of fact, an important unresolved point concerns the timing of the stroke rehabilitation: it is common practice to limit the intensive treatment to the first 3 months after the acute event and consider the subsequent rehabilitation proposals as maintaining the present functional conditions and prevent secondary complications. However, several studies offer evidence in support of a rehabilitation continuity in chronic hemiplegic patients (10–12) aimed at ensuring the achievement of further objectives in the long term, as well as maintaining the already achieved results in day hospital or rehabilitative outpatient setting. For example, it is essential to avoid the risk of falling in stroke patients, and therefore improve stability and balance (13, 14) even some time after the onset of symptoms. Neuronal plasticity plays an important role in motor recovery, as well as the equilibrium between excitatory and inhibitory signals in the brain pathways (15, 16).

Various processes such as restitution, substitution and compensation can explain how it is possible to notice some improvements in the recovery of the upper limbs even years after the acute event (17–20). Restitution means a reacquisition of the lost abilities; substitution refers to the replacement of the motor paths while motor compensation requires the adaptation of other motor elements (21). As an actual fact, international guidelines recommend a rehabilitation program continuation after discharge from the post-acute care center (22–24), lasting at least a few weeks up to months as required by residual impairment.

Different neurorehabilitation techniques are followed in chronic post-stroke patient, such as traditional physiotherapy, mirror therapy, Neuromuscular Electrical Stimulation (NMES), orthoses and robotic, virtual reality (VR). From the literature, it is often clear how neuro-rehabilitative exercise is often associated with innovative techniques such as VR or robotic training for gait recovery and it is not possible to indicate which technique or protocol is better than the other: there is a great heterogeneity in the protocols and setting (outpatient, at home, remotely in tele-rehabilitation).

At present time, an ideal neurorehabilitation approach among all the various therapy possibilities has not been established. The clinical and research question should be: “What is the best neuro-rehabilitative approach for patient with chronic stroke?” evaluating the resources of the social and family context too (presence or not of the care giver, accessibility to care).

Considering these premises, the following review lends itself to identify Randomized Controlled Trials (RCT) suggesting the possibility of achieving goals in rehabilitative interventions starting 6 months or more after the acute event, particularly regarding residual motor deficits.

Because the neuro-rehabilitative approach beyond the acute stroke event may not only have as its objective the maintenance of the motor function but the improvement of the function in social life.

The aim of this scoping review was to highlight the different treatment opportunities available in neurorehabilitation, effective for patients with chronic stroke sequelae, not only in terms of maintaining motor function but also improving it.

## 2. Materials and methods

### 2.1. Eligibility criteria

The Preferred Reporting Items for Systematic Reviews extension for Scoping Reviews (PRISMAScR) checklist was followed for writing this scoping review (25, 26).

All randomized controlled trials published from 2012 to January 2023 written in English and specifically dealing with the topic of “rehabilitation strategies in chronic hemiplegic patient after stroke” were considered as eligible if they reflected the following PCC framework: (i) Population: males and females with diagnosis of chronic stroke (≥ 6 months), age > 18 years old. (ii) Concept: rehabilitative techniques specifically addressed to treat motor impairment in chronic stroke patients according to International Guidelines (23). (iii) Context: sequelae, such as motor function, muscular strength, gait, postural balance, spasticity and quality of life, secondary to a chronic stroke.

We excluded pilot studies, studies without full-text available, without specified acute post-event period (6 months), with a PEDRO score under 5, (27) involving less than or equal to 10 patients, non-inferiority studies and studies which were not focused on the outcomes measures we meant to analyze.

### 2.2. Search strategy

A literature search was conducted (December 2022–January 2023) using the following databases: MEDLINE (PubMed), PEDro, Scopus, Web of Science (WOS), Cochrane. Keywords used were “chronic stroke AND rehabilitation, chronic stroke AND physiotherapy, chronic stroke AND recovery, chronic stroke AND exercise.” Searches were supplemented by hand searching of additional articles meeting eligibility criteria that were cited in reference lists.

### 2.3. Evidence screening and selection

All articles identified in the research were imported into Microsoft Excel. Two independent reviewers searched databases by using the same method to guarantee suitable cross-checking of the results. The authors evaluated the studies collected by the searches based on the inclusion and exclusion criteria established and selected them according with eligibility criteria. The authors independently checked the titles, abstracts, and full text of suitable studies.

### 2.4. Data extraction and analysis

Data extracted from selected studies were: Authors and date of study, type of interventions, outcomes, population, who delivered the intervention; study design and authors conclusions. All data required to answer the study questions were published in the articles. Any disagreements regarding the data collection were solved by discussion until consensus.

## 3. Results

### 3.1. Study selection

**Figure 1:**
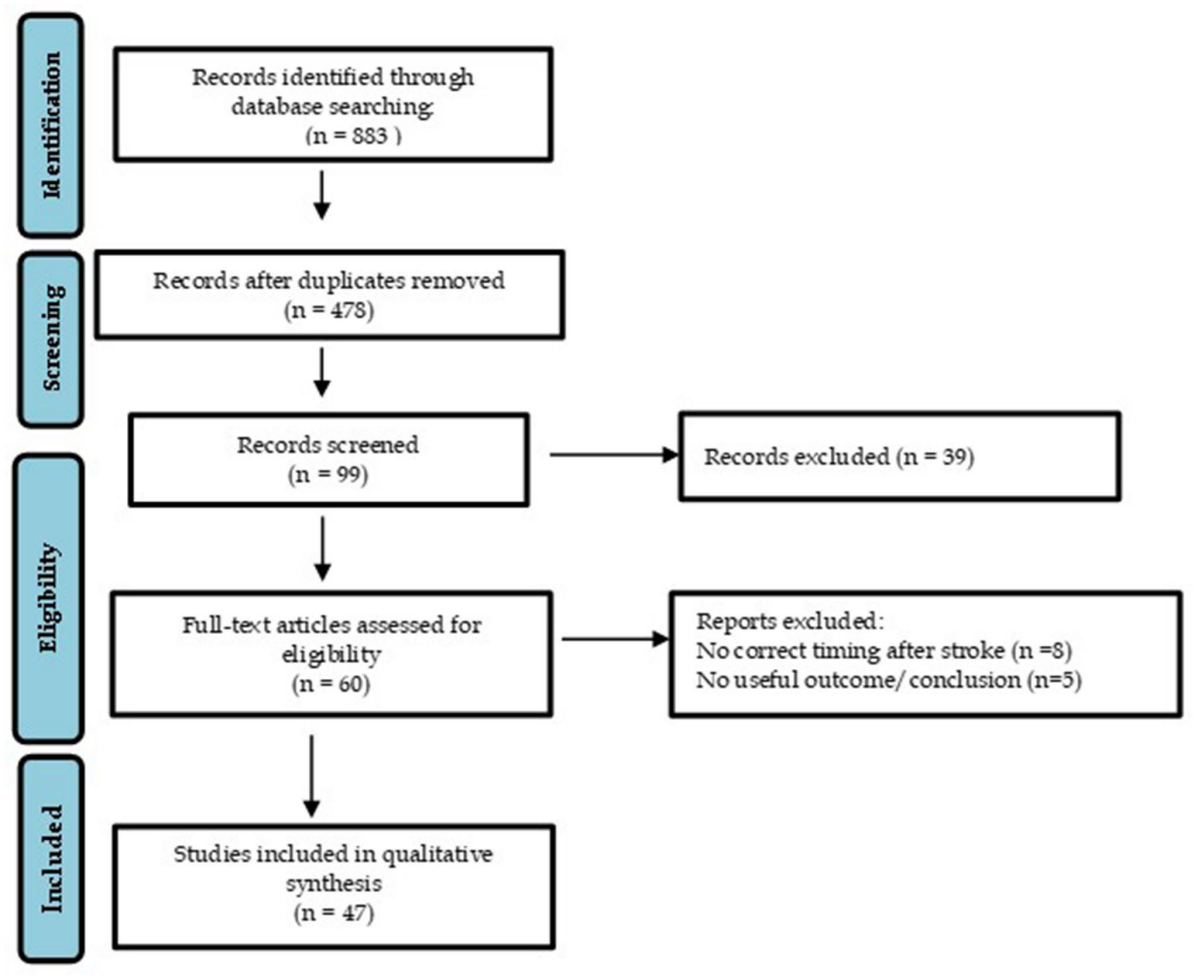
Flow chart.

As shown in the study flow chart (Figure 1) the literature search identified 883 records. After removing duplicates, the research resulted in 478 records. A total of 99 records were screened based on their titles and abstracts. Then 39 were discarded following application of the inclusion and exclusion criteria. Finally, 47 were considered relevant for qualitative analysis.

### 3.2. Characteristics of included studies

Characteristics of included studies are summarized in Table 1.

**Table 1:** General characteristics of included studies.

**Authors**	**Study population**	**Intervention type**	**Dosage and frequency of intervention**	**Outcome measures**	**Assessment intervals**	**Conclusions**	**Pedro scale**
Calabrò et al. (28)	40 patients allocated to: EGT group (Ekso Gait Training) (*n* = 20) OGT group (Overground Gait Training) (*n* = 20)	Ekso gait training	Patients underwent 60 min of conventional physiotherapy followed by 45 min of Ekso gait training or conventional gait training, five times/week for 8 weeks.	10 Meter Walk Test (10MWT), Rivermead Mobility Index (RMI), Timed Up and Go (TUG). Gait pattern (surface electromyography-sEMG from lower limbs), FPEC (frontoparietal effective connectivity) by using EEG, corticospinal excitability (CSE) and sensory-motor integration (SMI) by using transcranial magnetic stimulation paradigm over the affected and unaffected hemisphere.	T0 = baseline T1 = post-intervention	Ekso™ gait training seems promising in gait rehabilitation for post-stroke patients, besides OGT	7/10
Kooncumchoo et al. (29)	30 patients allocated to: I-Walk machine group (*n* = 15) overground gait training (control) group (*n* = 15).	I Walk machine: specific tool for assisting walking and controlling walking patterns (staircase walking) with an adjustable number of repetitive walking steps (0–120 steps/min)	30 min of upper limb and hand movement and sit-to-stand training. The experimental group received 30 min of I-Walk training, while the control followed a 30-min overground training program. All the individuals were trained 3 days/week for 8 weeks.	Fugl–Meyer Assessment (FMA), 6-Minute Walk Test (6 MWT), 10-Meter Walk Test (10 MWT), Timed Up and Go (TUG)	T0 = baseline T1 = 2 weeks T2 = 4 weeks T3 = 6 weeks T4 = post-intervention	The I-Walk training machine improves gait speed but slightly decreases the range of motion compared to conventional PT training	6/10
Wu et al. (30)	30 patients allocated to: Resistance group (*n* = 14) or, Assistance group (*n* = 14)	A custom-designed, cable-driven robotic gait training system used on a treadmill	Subjects trained 3 times a week for 6 weeks. Each training session was 45 min excluding setup time.	Self-selected and fast overground walking velocity on a 10-m instrumented walkway (GaitMat IIa), 6-Minute Walk Test (6MWT), Modified Ashworth Scale (MAS), Berg Balance Scale (BBS), Activities-specific Balance Confidence (ABC) Scale, Medical Outcomes Study 36-Item Short-Form Health Survey	T0 = baseline T1 = post-intervention T2 = 8-weeks follow up	Applying a controlled resistance or an assistance load to the paretic leg during treadmill training may induce improvements in walking speed in individuals post-stroke. Resistance training was not superior to assistance training in improving locomotor function in individuals post-stroke.	8/10
Lee et al. (31)	30 patients allocated to: experimental group (15), control group (15)	Afferent electrical stimulation (AES) combined with mirror therapy	Patients received 60 min sessions 5 times/week for 4 weeks	Handheld Dynamometer; Modified Ashwort Scale (MAS), Berg Balance Scale (BBS); GAITRite	T0 = baseline T1 = post-intervention	Mirror therapy with afferent electrical stimulation may effectively improve muscle strength and gait and balance abilities in hemiplegic stroke survivors	6/10
Arya et al. (32)	36 patients allocated to: experimental group (*n* = 19), control group (*n* = 17)	Activity-based MT comprised movements such as ball-rolling, rocker board, and pedaling. The activities were provided on the less-affected side in front of the mirror while hiding the affected limb.	Thirty sessions consisting of 1 hour each (3–4/week) were provided across the 3 months. The experimental group received MT protocol and conventional therapy for 30 min each. The control group was provided the conventional management for 1 h to match the dose	Brunnstrom Recovery Stages (BRS), Fugl-Meyer Assessment Lower Extremity (FMA-LE), Rivermead Visual Gait Assessment (RVGA), and 10-Meter Walking Test (10-MWT)	T0 = baseline T1 = post-intervention	Activity-based MT facilitates motor recovery of the lower limb as well as reduces gait deviations among chronic post-stroke hemiparetic subjects	8/10
Son et al. (33)	20 patients allocated to: self-observation training group (*n* = 10) or control group (*n* = 10)	Self-Observation training	30-min exercise therapy regimen, 5 days a week for 4 weeks. The self-observation training group additionally watched videoclips of their balance and functional gait training and performed physical training twice over a 10-min time span. Each self-observation training session was performed for 30 min, 3 times a week for 4 weeks	Surface Electromyography, Timed Up and Go (TUG), 10-Meter Walking Test (10MWT)	T0 = baseline T1 = post-intervention	Self-observation training improved lower limb muscle activity and dynamic balance in patients with chronic stroke	6/10
Bang et al. (34)	30 patients allocated to: action observational training group (*n* = 15), control group (*n* = 15)	Action Observational training (treadmill video)	Participants underwent training for 40 min per day, five times a week for 4 weeks.	Timed Up and Go Test (TUG), 10-Mt Walking Test (10MWT), 6-Minutes Walking Test (6MWT), maximal flexed knee angle in the swing phase during walking	T0 = baseline T1 = post-intervention	Action observational training is an effective method for improvement of the walking ability in chronic stroke patients	7/10
Cho et al. (35)	28 patients allocated to: experimental group (*n* = 15), control group (*n* = 13)	Motor imagery training was conducted using visual and kinematic imagery separately. Visual imagery is a process in which an individual imagines their physical movement from an external perspective, and kinematic imagery is a process in which an individual imagines internal sensory information during physical movement.	Imagery training was applied for 15 min, following gait training using a treadmill for 30 min. All interventions included gait training; imagery training was performed 3 times a week for 6 weeks.	Functional Reach Test, Timed Up-and Go Test (TUG), 10-mt Walking Test (10MWT) and Fugl-Meyer Assessment (FMA)	T0 = baseline T1 = post-intervention	Gait training with motor imagery training improves the balance and gait abilities of chronic stroke patients significantly better than gait training alone.	6/10
Dickstein et al. (36)	23 patients allocated to: experimental group (*n* = 12), control group (*n* = 11)	Integrated Motor imagery practice	The participants underwent 15-min sessions conducted 3 times a week for 4 weeks.	10-M Walking Test (10MWT), Falls Efficacy Scale Swedish version (FESS), Step Activity Monitor (SAM)	T0 = baseline T1 = post-intervention T2 = 2-weeks follow up	Home delivery of integrated motor imagery practice was feasible and exerted a positive effect on walking in the home	6/10
In et al. (37)	25 patients allocated to: VRRT group (*n* = 13), control group (*n* = 12)	Virtual reality reflection therapy (VRRT) on lower limbs	Participants received 30 min a day of conventional stroke rehabilitation program followed by 30 min of VRRT program or placebo/5 days a week for 4 weeks	Berg Balance Scale (BBS), Functional Reaching Test (FRT), Timed up and go (TUG), Balance system, 10 Mt Walking Test (10MWT)	T0 = baseline T1 = post-intervention	VRRT has beneficial effects on balance and gait ability in people with chronic stroke	5/10
Kayabinar et al. (38)	30 patients allocated to: VR + RAGT group (virtual reality + robot-assisted gait training, *n* = 15); RAGT (robot-assisted gait training, *n* = 15)	Virtual reality augmented added to robot-assisted gait training	The participants underwent a total of 12 sessions, 2 days a week for 6 weeks.	Functional Gait Assessment (FGA), Rivermead Mobility Index (RMI), Berg Balance Scale (BBS), The Fall Efficacy Scale International (FES-I), Functional Independence Measure (FIM)	T0 = baseline T1 = post-intervention	VR augmented RAGT improved dual-task gait speeds and dual-task performance of chronic stroke patients; however, there was no difference between the two groups after the treatment. Although functional improvements were determined with VR combined RAGT approach, it was not superior to RAGT only treatment	6/10
Llorens et al. (39)	20 patients allocated to: experimental group (*n* = 10), control group (*n* = 10)	Virtual reality-based exercise to train balance and postural control disabilities	20 one-hour sessions, 5 sessions per week. Experimental group combined 30 min with the virtual reality-based intervention with 30 min of conventional training. The control group underwent one hour of conventional therapy.	Berg Balance Scale (BBS), Tinetti Performance-Oriented Mobility Assessment, Brunel Balance Assessment (BBA), 10-m Walking Test (10MWT)	T0 = baseline T1 = post-intervention	Virtual reality interventions can be an effective resource to enhance the improvement of balance in individuals with chronic stroke	8/10
Alwhaibi et al. (40)	30 patients allocated to: intervention group (*n* = 15), control group (*n* = 15)	Standard physical therapy plus somatosensory stimulation	Participants underwent 3 treatments/week for 8 weeks. The control group received 4 components of standard physical therapy program (25 min—15 min—10 min—10 min) while the control group received the same exercises but with a different duration (15 min—10 min—5 min—5 min). Patients also received thermal stimulation (TS) training.	Functional Independent Measure (FIM) and Quantitative Electroencephalography (QEEG)	T0 = baseline T1 = post-intervention	TS is one of the advanced approaches in rehabilitation and may improve functional performance of the affected lower extremity and neural activity of the brain.	6/10
Yang et al. (41)	25 patients allocated to: NMES-TA group (Tibialis Anterior, *n* = 8); NMES MG group (Medial Gastrocnemius, *n* = 9); control group (*n* = 8)	NMES protocol on plantar and dorsiflexors muscles	The experimental groups received 20 min-sessions of NMES. The control group received 20 min of range of motion and stretching exercises. After NMES or exercises, all participants received ambulation training for 15 min. Training sessions occurred 3 times per week for 7 weeks.	GAITRite: gait velocity, cadence, step length. Modified Ashworth Scale (MAS), EMG ankle plantar flexors during gait, handheld dynamometer, maximum position of the ankle joint during gait	T0 = 7 days before the first session T1 = after 21 sessions	Applying NMES on ankle dorsiflexors might be an effective strategy for muscle strengthening and spasticity reduction to enhance ankle control during push off and gait performance. Also, it could result in favorable effects on temporal gait symmetry in chronic stroke individuals with inadequate ankle control.	6/10
Bethoux et al. (42)	399 subjects allocated to: WA group (Walk-Aid, *n* = 187), AFO group (Ankle- Foot Orthosis, *n* = 212)	Peroneal nerve functional electrical stimulation (FES) as an alternative to ankle-foot orthoses (AFO)	Patients wore WalkAide FES system (WA) or an AFO for 6 months.	10MWT, Stroke Impact Scale (SIS), 6MWT, GaitRite, Functional Ambulation Profile (FAP), Modified Emory Functional Ambulation Profile (mEFAP), BBS, TUG, Stroke-Specific Quality of Life (SSQoL), and individual SIS domain scores	T0 = baseline T1 = post-fitting, with device T2 = 1 month, T3 = 3 months T4 = 6 months	Use of FES is equivalent to the AF	5/10
Beaulieu et al. (43)	18 patients allocated to: RPMS group (*n* = 9), sham group (*n* = 9). healthy subjects (*n* = 14).	Repetitive Peripheral Magnetic Stimulation (RPMS)	The participants received the intervention (RPMS) in a single session lasting 2-3 hours.	Ankle range dorsiflexion (ROM); isometric muscle strength of dorsiflexor muscles; resistance of plantar flexors to stretch; TMS (transcranial magnetic stimulation) testing	T0 = baseline T1 = post-intervention	RPMS improved ankle impairments in chronic stroke patients	5/10
Lee et al. (44)	31 patients allocated to: local vibration stimulus training program group (*n* = 16) and sham group (*n* = 15)	Local Vibration stimulus training program	Participants underwent to training session for 30 min a day, five times a week, for 6 weeks	Balance, GAITrite	T0 = baseline T1 = post-intervention	Local vibration stimulus training program is an effective method for improvement of the postural sway and gait ability of chronic stroke patients	7/10
Park et al. (45)	30 patients allocated to: TENS Group + therapeutic exercise (*n* = 15), Placebo TENS Group + therapeutic exercise (*n* = 15)	TENS associated with therapeutic exercise	Patients underwent sessions of 30 min for 5 days/ week, lasting 6 weeks	Modified Ashworth Scale (MAS), balance system, Timed Up and Go (TUG), Gait analyzer	T0 = 1 week before treatment T1 = 1 week after treatment	A combination of therapeutic exercise and TENS may reduce spasticity and improve balance, gait, and functional activity in chronic stroke patients	6/10
Lim et al. (46)	17 patients allocated in: HBP group (home-based rehabilitative program, *n* = 9), control group (*n* = 8)	Home-based rehabilitative programs on postural balance	Participants received the treatment five times per week for 6 weeks.	10 Mt Walking Test (10MWT), Figure of 8 walk test, Four-square step test, 36 item Short-Form Survey (SF-36)	T0 = baseline T1 = post-intervention	HBP group received positive benefits with regard to the postural balance and walking abilities compared to the clinical setting exercise program	6/10
Hornby et al. (47)	90 patients allocated to: high variable protocol group (*n* = 28), high forward protocol group (*n* = 30), low variable protocol group (*n* = 32)	High-intensity stepping of variable stepping tasks (high variable), high-intensity stepping performing only forward walking (high forward), and low-intensity stepping in variable contexts at 30–40% heart rate reserve (low variable)	Participants received ≤ 30 one-hour training sessions over 2 months (3–5 sessions/wk), with ≤40 min of stepping practice each session.	6 Minutes Walking Test (6MWT), Functional Gait Assessment, 5-times sit-to-stand Test	T0 = baseline T1 = post-intervention T2 = 3-months FU	High-intensity stepping training resulted in greater improvements in walking ability and gait symmetry than low-intensity training in individuals with chronic stroke, with potential greater improvements in balance confidence	8/10
Globas et al. (48)	38 patients allocated to: TAEX group (*n* = 20), control group (*n* = 18)	Progressive graded, high-intensity aerobic treadmill exercise (TAEX)	Treadmill training (TAEX) consisted of 39 sessions (3×/week; 3 months) vs usual care physiotherapy (control) according to the typical German prescription (1–3 sessions/week)	6-min walking test, 10-Mt Walking Test (10MWT), 5-Chair-Rise (5CR) test, Berg Balance Scale (BBS), Rivermead Mobility Index (RMI), and Medical Outcomes Study Short-Form 12 (SF-12)	T0 = baseline T1 = 3 months post-training T2 = 12 months follow up	Aerobic treadmill exercise in chronic stroke survivors improves cardiovascular fitness, gait, balance, mobility and quality of life	7/10
Chen et al. (49)	30 participants allocated to: experimental group (*n* = 15), control group (*n* = 15)	Rotational treadmill was designed to provide turning-based treadmill training	Participants underwent 12 sessions of 40 min over 4 weeks	GAITtrite, Turning Performance, LOS, Sensory Organization Test (SOT), Handheld Dynamometer, Berg Balance Scale (BBS)	T0 = baseline T1 = post-intervention T2 = 1 month follow up	Turning-based treadmill training may be a feasible and effective strategy to improve turning ability, gait symmetry, muscle strength, and balance control for individuals with chronic stroke	7/10
Choi et al. (50)	30 patients allocated to: WBV-TT group (whole body vibration—treadmill training, *n* = 15), treadmill training group (TT, *n* = 15)	Whole-body vibration combined with treadmill training	WBV-TT was performed 3 times a week (4.5 min per session) for 6 weeks. Each session included 6 exercises and each exercise was conducted for 45 seconds	Gait-rite, 6m-wt	T0 = baseline T1 = post-intervention	WBV-TT is more effective than TT for improving walking performance of patients with chronic stroke	8/10
Cho et al. (51)	30 allocated to: TRWVR group (treadmill training based real-world video recording, *n* = 15), control group (*n* = 15)	Treadmill training based real-world video recording (TBRVR)	Patients underwent training sessions of 30 min per day, three times per week, for 6 weeks	Dynamic and Static balance, Berg Balance Scale (BBS), Timed Up and Go (TUG), GAITrite	T0 = baseline T1 = post-intervention	Real-world video recording influences dynamic balance and gait in chronic stroke patients when added to treadmill walking	7/10
Hwang et al. (52)	32 participants allocated to: Treadmill training with Tilt Sensor FES (TTSF) group (*n* = 16) and Treadmill training with Placebo Tilt Sensor FES (TPTSF) group (*n* = 16).	TTSF group performed gait training on treadmill with tilt sensor FES, and TPTSF group performed gait training on treadmill with placebo tilt sensor FES.	Treadmill training combined with FES was performed for 30 min, one time per day, for 4 weeks. Conventional physical therapy was performed for 30 min, twice per day, for 4 weeks.	10-Mt Walking Test (10MWT), Berg Balance Scale (BBS), Timed Up-and Go (TUG)	T0 = baseline T1 = post-intervention	TTSF can be an effective intervention for improving balance, gait ability, and muscle architecture of tibialis anterior of stroke survivors	7/10
An et al. (53)	26 participants allocated to: MWM group (Mobilization with Movement, *n* = 13) and control group (*n* = 13)	Talocrural MWM (mobilization with movement) administered by a physiotherapist	Both groups attended conventional physiotherapy sessions 3 times a week for 5 weeks (30 min per session). Additionally, the MWM group underwent talocrural MWM 3 times a week for 5 weeks	Korean version of the Modified Barthel Index (K-MBI), Modified Ashworth Scale (MAS), Maximal concentric contraction measured with an isokinetic dynamometer	T0 = baseline T1 = post-intervention	Talocrural MWM has an augmented effect on ankle strength, mobility, and weight-bearing ability in chronic stroke patients with limited ankle motion when added to conventional therapy	6/10
Park et al. (54)	38 patients allocated to: S-MWM group (self- mobilization with movement, *n* = 19) and CMS groups (calf mus- cle stretching training, *n* = 19)	The participants themselves performed the CMS. While in a lunge position (the affected side leg is forward), participants conducted S-MWM with a non-elastic strap approximately 40 cm long.	Conventional physiotherapy for 30 min per session + S-MWM and CMS techniques performed 3 times per week for 4 weeks	Ankle DF-PROM, GAITRite system, Biodex Balance System	T0 = baseline T1 = post-intervention	Both groups showed significant improvement in all outcome measures; ankle DF-PROM, gait parameters (gait speed, cadence, and stride lengths on both sides), and fall risk showed greater improvement in the S-MWM group than in the CMS group	7/10
Liao et al. (55)	56 patients allocated to: Balance Training group (*n* = 19); Lateral Wedge group (*n* = 18); Control group (*n* = 19)	The BT group received the weight shift training using the Biodex Balance System, as well as visual biofeedback balance training. The LW group used a 5° lateral wedge insole placed in the shoe of their healthy side for usual standing and walking.	BT group: the patients received 20 min of training 3 times/week for 6 consecutive weeks	CAT (balance computerized adaptive test), TUG test (Timed Up and Go)	T0 = baseline T1 = post-intervention T2 = 10 weeks follow up T3 = 18 weeks follow up	Six-week visual biofeedback training and intervention of 5° lateral wedge insoles can improve the balance ability of patients with a chronic stroke	8/10
Cho et al. (56)	38 patients allocated to: RT-AAN group (Robot-assisted reach training with assist-as-needed, *n* = 19) RT-G group (robot-assisted reach training with guidance force, *n* = 19)	Robot-assisted reach training with assist-as-needed (RT-AAN)	All participants underwent the training program 3 times a week for 6 weeks. A single training session lasted 40 min.	Fugle Meyer Assessment (FMA), Action Research Arm Test (ARAT) and Box and Block Test (BBT)	T0 = baseline T1 = 3 days after the end of intervention.	RART (robot-assisted reach training) with an active assistant protocol showed improvements of upper extremity function and kinematic performance	8/10
Cordo et al. (57)	43 patients allocated to: Torque group (*n* = 22), EMG group (*n* = 21)	Assisted movement and muscle vibration combined with either torque or EMG biofeedback	Each participant received 30 sessions (30 min duration per session) directed at the impaired hand over 10–12 weeks.	Upper Extremity-Fugl Meyer Assessment (UE-FMA), Strength Test, Box and Block Test (BBT) and Stroke Impact Scale (SIS)	T0 = baseline T1 = post-intervention	Assisted movement and muscle vibration, combined with EMG or torque biofeedback, appears to reduce upper limb impairment, improve volitional activation of the hand muscles and restore a modicum of hand function	5/10
Hung et al. (58)	44 patients allocated to: UHT group (unilateral hybrid therapy, *n* = 14), BHT group (bilateral hybrid therapy, *n* = 15), RT group (robot-assisted therapy, *n* = 15).	UHT combined unilateral RT (URT) and modified constraint-induced therapy. BHT combined bilateral RT (BRT) and bilateral arm training	The RT group received URT and BRT. The intervention frequency for the three groups was 90 min/day, 3 days/week for 6 weeks.	Fugl-Meyer Assessment (FMA), Stroke Impact Scale (SIS), Wolf Motor Function Test (WMFT) and Nottingham Extended Activities of Daily Living (NEADL) scale	T0 = baseline T1 = post-intervention T2 = 3 months follow up (FMA and SIS scores)	BHT was more effective for improving upper extremity motor function, particularly distal motor function at follow-up, and individuals in the RT group demonstrated improved functional ambulation post-intervention.	8/10
Lin et al. (59)	33 patients allocated to: bilateral isometric handgrip force training group (*n* = 16), control group (*n* = 17)	The computer-aided interlimb force coupling training task with visual feedback included different grip force generation methods on both hands.	The bilateral isometric handgrip force training consisted of 30 min of training 3 days per week for 4 weeks, for a total of 12 sessions.	Barthel Index (BI), Upper Extremity Fugl-Meyer Assessment (FMA-UE), Motor Assessment Score (MAS) and Wolf Motor Function Test (WMFT)	T0 = baseline T1 = post-intervention	Computer-aided interlimb force coupling training improves the motor recovery of a paretic hand and facilitates motor control and enhances functional performance in the paretic upper extremity	7/10
Choi et al. (60)	36 patients allocated to: Gesture Recognition (GR) Mirror Therapy group (*n* = 12), Conventional Mirror Therapy group (*n* = 12), Control group (*n* = 12)	Gesture Recognition Mirror therapy	The patients received 15 intervention sessions of 30 min/day, 3 days/week for 5 weeks.	Manual Function Test (MFT), Neck Discomfort Score (NDS), 8-Item Short-Form Health Survey (SF-8)	T0 = baseline T1 = post-intervention	GR mirror therapy has a positive effect on upper-extremity motor function and quality of life. Traditional mirror therapy produces less neck discomfort.	7/10
Colomer et al. (61)	31 patients allocated to: experimental group (*n* = 15); control group (*n* = 16)	Mirror Therapy program included in a physical therapy program focused on balance and training	Patients underwent 24 training session of 45 min for 3 times a week	Wolf Motor Function Test (WMFT), Upper Limb motor function (FMA), Nottingham Sensory Assessment (NSA)	T0 = baseline T1 = post-intervention	MT in chronic stroke survivors with severely impaired upper limb function may provide a limited but positive effect on light touch sensitivity while providing similar motor improvement	8/10
Cho et al. (62)	27 patients allocated to: experimental group (*n* = 14) and a control group (*n* = 13)	Transcranial direct current stimulation (tDCS) matched with mirror therapy (MT)	tDCS for 20 min (2 mA intensity) followed by a 5 min rest. The experimental group received MT while the control group conducted the same exercises as the experimental group using a mirror that did not show the non-paretic upper extremity. The groups performed the same exercises for 20 min. All subjects received this intervention for 45 min three times a week for 6 weeks	Box and Block test (BBT), Grip Strength, Fugl-Meyer Assessment (FMA), Jebsen-Taylor Test	T0 = baseline T1 = post-intervention	MT with tDCS has a positive effect on the functional recovery of the upper extremity of stroke patients	5/10
Hernandez et al. (63)	51 participants allocated to: treatment group (*n* = 26) or standard care group (*n* = 25)	Use of the Jintronix system as a remotely supervised home-based program for UE rehabilitation	Each intervention consisted of a 4-week long program. Experimental group: home-based exercise program via the Jintronix system monitored offline by a therapist. Control group: home-based exercise program manual (Graded Repetitive Arm Supplementary Program).	Fugl-Meyer Assessment for UE (FMA-UE), Stroke Impact Scale (SIS), Motor Activity Log-14	T0 = baseline T1 = post-intervention T2 = 4 weeks follow up	These findings suggest that UE training for chronic stroke survivors using virtual rehabilitation in their home may be as effective as a gold standard home exercise program and that those who used the system the most achieved the greatest improvement in UE	8/10
De Diego et al. (64)	21 patients allocated to: experimental group (EG, *n* = 12), control group (CG, *n* = 9)	Conventional rehabilitation therapy according to the Bobath concept and sensory and motor stimulation of the upper limb	The EG received 16 sessions of the protocol of 1 hour at the center for 8 weeks, 2 sessions per week, and 1 daily session of 30 min of functional activity training at home. The CG had the usual treatment according to the Bobath concept, without prioritizing therapy of the upper limb, with 2 sessions per week.	FMA, Motor Activity Log Amount Scale (MAL—AS), Motor Activity Log How Well (MAL- HW), Stroke Impact Scale 16 (SIS-16)	T0 = baseline T1 =after 8 sessions T2 = post-intervention	The intensive sensorimotor stimulation program for the upper extremity may be an efficacious method for improving function and use of the affected limb in ADL in chronic stroke patients	6/10
Lee et al. (65)	39 patients allocated to: RT combined with NMES group (RT + ES, *n* = 20), RT with sham stimulation group (RT + Sham, *n* = 19)	Bimanual RT (robot therapy) combined with NMES (Neuromuscular Electrical Stimulation)	The participants received their respective interventions for 20 training sessions (90–100 min/day, 5 days/week for 4 weeks)	Upper Extremity Fugl-Meyer Assessment (UE-FMA), Modified Ashworth Scale (MAS), Wolf Motor Function Test (WMFT), Motor Activity Log (MAL), and Stroke Impact Scale 3.0 (SIS)	T0 = baseline T1 = post-intervention T2 = 3 months FU	RT + ES induced significant benefits in reducing wrist flexor spasticity and in hand movement quality	7/10
Knutson et al. (66)	80 patients allocated to: CCFES group (*n* = 40), NMES group neuromuscular electrical stimulation (*n* = 40).	Contralaterally controlled functional electrical stimulation (CCFES)	CCFES and NMES treatments lasted 12 weeks and consisted of: (a) 20 sessions of therapist-guided functional task practice in the lab (two per week except on weeks that included an assessment session), (b) 10 sessions per week of self-administered repetitive hand opening exercise at home. Functional task practice (FTP) was performed for 60 min per session.	Box and Blocks Test (BBT) score, Upper extremity Fugl-Meyer (UEFM) and Arm Motor Abilities Test (AMAT)	T0 = baseline, T1-T2-T3 = every 3 weeks during the treatment period, T4 = end of treatment, T5-T6-T7: 2, 4, and 6 months after end of treatment	CCFES improved hand dexterity more than NMES in chronic stroke survivors.	6/10
Tavernese et al. (67)	44 patients allocated to: experimental group (*n* = 24), control group (*n* = 20).	Segmental Muscle Vibration (SMV)	All the participants underwent a 60-min general physical therapy session, 5 times per week, over a period of 2 weeks. Participants in the EG also received 30 min of SMV therapy (120 Hz).	Normalized jerk (NJ), mean linear velocity (ms^−1^); movement duration (s); movement length (m); HTD (hand target distance) at the end of movement (m); mean angular velocity at the shoulder (°s^−1^); angle at the elbow at the end of movement (°)	T0 = baseline T1 = 2 weeks post-intervention	A combined treatment of SMV and therapeutic exercise determines a significant improvement of motor performance in the paretic upper limb during reaching movement	8/10
Costantino et al. (68)	32 patients allocated to: group A treated with vibration protocol (*n* = 17); group B with sham therapy (*n* = 15)	Local muscle vibration	Application of local muscle vibration set to a frequency of 300 Hz, for 30 min 3 times per week, for 12 sessions, applied to the skin covering the venter of triceps brachii and extensor carpi radialis longus and brevis muscles during voluntary isometric contraction	Hand Grip Strength Test, Modified Ashworth Scale (MAS), QuickDASH score, FIM scale, Fugl-Meyer Assessment (FMA), Jebsen-Taylor Hand Function Test and Verbal Numerical Rating Scale of pain	T0 = baseline T1 = post-intervention	Rehabilitation treatment with local muscle high frequency (300 Hz) vibration for 30 min, 3 times a week for 4 weeks, could significantly improve muscle strength and decrease muscle tonus, disability and pain in upper limb of hemiplegic post-stroke patients.	6/10
Karthikbabu et al. (69)	85 patients allocated to: Plinth group (*n* = 30), Swiss ball group (*n* = 28), Control groups (*n* = 27)	Selective upper and lower trunk movements using either plinth or Swiss ball	Patients in the experimental groups received selective upper and lower trunk movements in supine and sitting positions using either stable support (plinth) or unstable support (Swiss ball). Patients underwent 1 hour exercise session, 3 sessions per week over a duration of 6 weeks.	Trunk impairment Scale, Brunel balance assessment, Tinetti scale, reintegration to normal living index, 10m-wt	T0 = baseline T1 = post-intervention T2 = 3 months follow up T3 = 12 months follow up	Plinth and Swiss ball-based trunk exercise regimes showed significant improvements in balance, mobility, physical function, and community reintegration in chronic stroke as against standard physiotherapy	7/10
Lee et al. (70)	28 patients allocated to: CCS group (Conventional Core Stabilization, *n* = 14), DNS group (Dynamic Neuromuscular Stabilization, *n* = 14)	CCS Conventional core stabilization DNS Dynamic neuromuscular stabilization	Both groups received a total of 20 sessions of CCS or DNS training for 30 min per session, 5 times a week during the 4-week period	Electromyography was used to measure the APA time for bilateral external oblique (EO), transverse abdominis/internal oblique (TrA/IO), and erector spinae (ES) activation during rapid shoulder flexion. Trunk Impairment Scale (TIS), Berg Balance Scale (BBS), Falls Efficacy Scale (FES)	T0 = baseline T1 = post-intervention T2 = 2 years follow up	Core stabilization exercises improve APA control, balance, and fear of falls in individuals with hemiparetic stroke.	5/10
Lee et al. (71)	28 patients allocated to: dual motor task training group (*n* = 14) and control group (*n* = 14)	Dual motor task exercises	Conventional exercise program for 60 min per day, 5 times a week for 6 weeks. The dual motor task training group also performed dual motor task training in the sitting position for 30 min per day, 3 times a week for 6 weeks	Trunk impairment scale (TIS), modified functional reach test (MFRT)	T0 = baseline T1 = post-intervention	Dual motor task training combined with a conventional exercise program improves trunk control ability and sitting balance	5/10
Park et al. (72)	29 patients allocated to: LATE group (*n* = 14); control Group (*n* = 15)	LATE program: land-based and aquatic exercises for trunk control and performed in the supine and sitting positions	Both groups received neurodevelopmental treatment (Bobath approach) for 30 min/day, 5 days/week, for 4 weeks. In addition, the LATE group performed the LATE program for 30 min/day, 5 days/week, for 4 weeks.	Trunk Impairment Scale (K-TIS), 5-item, 3-level Postural Assessment Scale for Stroke (PASS-3L), 3-level Berg Balance Scale (BBS -3L), Functional Reach Test (FRT), Modified Barthel Index (MBI)	T0 = 3 days before treatment T1 = post-intervention	LATE program can help improve trunk control, balance, and activities of daily living in chronic stroke patients and may be used as a practical adjunct to conventional physical therapy	7/10
Pérez-de la Cruz (73)	41 patients allocated to: control group—dry land therapy (*n* = 15), experimental group − aquatic therapy (*n* = 13), combined group − aquatic + dry land therapy (*n* = 13)	Aquatic therapy program influence on chronic stroke patients	The participants trained for a 12-week period: the sessions lasted 45 min and were conducted twice weekly.	VAS, resilience scale, 36-Item Short-Form Health Survey (SF36)	T0 = baseline T1 = post-intervention T2 = one month follow up	Physical exercise performed in water has positive effects on several factors that contribute toward improving the mood and quality of life of people with acquired brain injury	8/10
Ku et al. (74)	20 patients allocated to: experimental group (*n* = 10), control group (*n* = 10)	Ai Chi (water-based exercise emphasizing characteristics of balance training)	The training session lasted 60 min/time, 3 times/week for a total of 6 weeks	LOS, BBS; GAITRITE, FMA	T0 = baseline T1 = post-intervention	Ai Chi is feasible for balance training in stroke and is able to improve weight shifting in anteroposterior axis, functional balance, and lower extremity control as compared to conventional water-based exercise	8/10

The included studies were heterogeneous because they have treated different rehabilitative techniques. Following our inclusion criteria, in our research we focused on various domains such as function, balance, walking abilities, spasticity and quality of life. Later we decided to analyze these RCTs dividing them in three different groups based on the principal aim: lower limb, upper limb and other outcomes; then we settled the studies in subgroups by the used rehabilitative technique. The mean and standard deviation values of the studies Pedro Score were, respectively, 6.68 and 1.04.

#### 3.2.1. Lower limb recovery

##### 3.2.1.1. Robotic technology

In Calabrò et al. work, the protocol with Ekso, an exoskeleton, showed a significant improvement at 10 Meter Walking Test (10MWT), Cortico-Spinal Excitability (CSE) and Sensory-Motor Integration (SMI) in the affected side, overall gait quality, hip and knee muscle activation and Frontoparietal Effective Connectivity (FPEC) (28). Similar results in walking speed were seen in Kooncumchoo et al. study where they compared an innovative end effector robot (I-Walk) to overground gait training (29). Instead, Wu et al. analyzed if there were differences between a robotic resistance training and a robotic assistance training. The results were similar except for better improvements in 6 Meter Walking Test (6MWT) and Berg Balance Scale (BBS) in the assistance training (30).

##### 3.2.1.2. Mirror neuron system

Lee et al. investigated how afferent electrical stimulation associated with mirror therapy could modify various outcomes in chronic stroke patients’ life. After 4 weeks, important differences were found in Berg Balance Scale (BBS), gait velocity, step length and stride length (31). In Arya et al. work, an activity-based mirror therapy was compared to a conventional one; after 3 months of intervention, there were statistically significant improvements in Fugl Meyer Lower Extremity (FMA-LE), Rivermead Visual Gait Assessment (RVGA) and Brunnstrom recovery stages (BRS-LE). No meaningful one was found at 10MWT (32). In Son et al. study, the experimental group performed a self-observation training associated with exercise therapy with significant ameliorations in muscle activity of the rectus femoris, biceps femoris, tibialis anterior and gastrocnemius and an improvement in 10MWT and Timed Up and Go (TUG) (33). The intervention group in Bang et al. paper watched a video of treadmill walking actions taken at various speeds before the training itself with significant progress in TUG, 10MWT, 6MWT and in maximal flexed knee ankle in the swing phase during walking (34).

##### 3.2.1.3. Motor imagery

Cho et al. investigate how motor imagery training with gait training, compared to classic gait training, could modify balance and gait abilities. After intervention there were significant improvements in FRT (Functional Reach Test), TUG (Timed Up-and-Go) and 10MWT (35). Dickstein et al. used a motor imagery technique with motor and motivational contents performed in the participants’ home with a significant amelioration concerning In-home walking (36).

##### 3.2.1.4. Virtual reality

In et al. investigated the effects of virtual reality reflection therapy (an enhanced version of the mirror therapy concept) on chronic stroke patients. After 4 weeks of program, there were significant improvements in the experimental group in BBS, Functional Reach Test (FRT), TUG, postural sway and 10MWT (37). In Kayabinar et al. study, virtual reality augmented robot-assisted gait training was compared to conventional robot-assisted gait training; after 4 weeks of intervention, there were ameliorations in motor task and cognitive task added to 10MWT in cognitive dual-task, although there was no statistically significant difference with the control group. FGA, RMI, BBS, and Functional Independence Measure (FIM) total scores improved in both groups but with no differences (38). In Llorens et al. study, a virtual reality-based stepping training was used in the intervention group to see any improvement in balance. After 20 sessions, there were significant changes in BBS, 10MWT and in Brunel Balance Assessment (BBA) (39).

##### 3.2.1.5. Somatosensory training

Alwhaibi et al. evaluated the effects of somatosensory rehabilitation on neural and functional recovery of Lower Extremity. After treatment, there was a significant improvement in FIM but no significant changes were found in QEEG scores (40).

##### 3.2.1.6. Electrical and repetitive peripheral magnetic stimulation

In Yang et al. study, Neuro-Muscular Electrical Stimulation (NMES) on anterior tibialis muscle or medial gastrocnemius muscle was compared to range of motion and stretching exercises in patients with inadequate ankle control. After 7 weeks of training, the NMES-TA group showed significant improvements in dynamic spasticity, spatial asymmetry, ankle plantarflexion during push off and muscle strength of ankle dorsiflexors (41). In Bethoux et al. work, the aim was to compare the effects of peroneal nerve Functional Electrical Stimulation (FES) to Ankle-Foot Orthosis (AFO). After 6 months of intervention the results were that FES stimulation is equivalent to AFO with no significant differences between groups (42). Beaulieu et al. used Repetitive Peripheral Magnetic Stimulation (RPMS) on paretic tibialis anterior muscle with a significant increase in ankle dorsiflexion mobility and maximal isometric strength and a decrease in resistance to plantar flexor stretch (43).

##### 3.2.1.7. Local vibration stimulus program

In Lee et al. study, a local vibration stimulus training program was applied to see the effects on postural sway and gait with significant results, after 6 weeks, in postural sway distance with eyes-open and closed, in postural sway velocity with eyes-open and closed and in gait speed, cadence, step length and single limb support time (44).

##### 3.2.1.8. Physical exercises

Park et al. associated a two-channel TENS, placed on the affected lower extremity on lateral and medial quadriceps muscle and gastrocnemius muscle, with an exercise program. After 6 weeks there were significant improvements in spasticity, static balance parameters, dynamic balance, gait speed and cadence, step and stride length on the paretic side (45). Lim et al. investigated the effects of a home- based rehabilitation program on postural balance, walking and quality of life with ameliorations in postural balance, comfortable speed, and fast speed walking but with no significant differences with the control group (46). Hornby et al., with their study, underlined how a high-intensity training can bring significant improvements in stepping amount and rate, with additional gains in spatiotemporal symmetry and balance confidence (47).

##### 3.2.1.9. Treadmill training

Globas et al. measured how high-intensity aerobic treadmill training influenced gait performances with a significant progress of peak exercise capacity and 6MWT in the intervention group, maintained also at 1-year follow up with a little decrease of walking capacities (48). In Chen I et al. work, a turning-based treadmill was compared to a normal treadmill, and after 4 weeks of training they noticed significant improvements on turning speed, straight-walking performance, strength of hip muscles and ankle dorsiflexors and balance control, maintained also at the 1-month follow up (49). In Choi et al. study, whole-body vibration was combined with treadmill training with significant changes in walking performances, gait parameters and 6MWT compared to control group (50). Cho et al. matched treadmill training with a real-world video recording; after 6 weeks there were significant improvements in dynamic balance and gait parameters (51). On the other hand, Hwang D. et al. used tilt sensor functional electrical stimulation on common peroneal nerve in treadmill training with significant ameliorations in TUG, BBS, 10MWT and anterior tibialis muscle architecture in the intervention group (52).

##### 3.2.1.10. Other types of intervention

An et al. investigated how a talocrural mobilization with movements can influence ankle strength, dorsiflexion passive range of motion and weight-bearing ability on the paretic limb: they showed significant ameliorations compared to the control group after 5 weeks of intervention (53). In Park et al. work, the intervention group experienced a four-week training of self-ankle mobilization with movement, and it was compared to a calf muscle stretching group; significant changes were found in gait parameters and fall risk (54). On the other hand, Liao W. et al. put in evidence how lateral wedge soles and visual biofeedback balance training group can improve balance Computerized Adaptive Test (CAT) and TUG test (55).

#### 3.2.2. Upper limb recovery

##### 3.2.2.1. Robotic technology

Cho et al. used an upper limb exoskeleton machine with two different protocols: the robot-assisted as needed protocol was significantly more effective than the robot-assisted with guidance force protocol in FMA and Action Research Arm Test (ARAT) (56). Cordo et al. analyze if there are significant differences between the use of EMG biofeedback and torque biofeedback in robot-assisted movement associated with muscle vibration in severe hand impairment following stroke but the results showed no meaningful distinction between them (57). Hung et al. investigate if a hybrid approach (arm training + robot therapy) could bring changes in motor function. They divided their patients in three groups: unilateral hybrid RT, bilateral hybrid RT and robot assisted therapy. The results favored Bilateral Hybrid Therapy (BHT) over Unilateral Hybrid Therapy (UHT) on the FMA total score and distal score. RT group showed significant improvements in the mobility domain of Nottingham Extended Activities of Daily Living (NEADL) (58). Lin et al. investigated the effects of a computer-aided bilateral isometric handgrip on paretic hand and arm motor control in chronic stroke patients. After 4 weeks of interventions, there were significant improvements in the FMA-UE, BI (Barthel Index), Wolf Motor Function Test (WMFT) and Modified Ashworth Scale (MAS) (59).

##### 3.2.2.2. Mirror neuron system

In Choi et al. work, mirror therapy was associated with a Gesture Recognition device (GR group) and compared with a second group with conventional Mirror Therapy (MR group) and a third group with sham therapy (CG). There were significant changes in upper extremity function, depression, and quality of life in the GR group and improvements in neck discomfort in MR and CG group (60). Lee et al. investigated the effects of afferent electrical stimulation associated with mirror therapy in stroke patients’ life: there was an increase in muscle strength measured with a handheld dynamometer (31). In Colomer et al. study, mirror therapy was applied to chronic stroke patients with severely impaired upper limb function. After 8 weeks, the experimental group showed significant improvements in tactile sensation compared to the control group but a similar increase in FMA and ability subscales in WMFT (61). Cho et al. associated mirror therapy with transcranial Direct Current Stimulation (tDCS). This innovative technique showed significant improvements compared to control group in the Box and Block Test (BBT) and grip strength (62).

##### 3.2.2.3. Virtual reality technique

In Hernandez et al. work, a virtual reality- based rehabilitation was compared to an evidence-based home exercise program in influencing upper extremity function; no significant differences were found between groups (63).

##### 3.2.2.4. Somatosensory training

De Diego et al. compared a sensory stimulation and functional activity training with a control group who went under a standard rehabilitation program. After 8 weeks there were significant improvements in the experimental group especially in the sensory tests (64).

##### 3.2.2.5. Electrical stimulation

Lee et al. decided to investigate the effects of combining robot-assisted therapy with neuromuscular electrical stimulation; after 4 weeks of intervention there were significant changes in wrist flexors MAS score, WFMT quality of movement and the hand function domain of Stroke Impact Scale (SIS) (65). Knutson et al. compared Contralaterally Controlled Functional Electrical Stimulation (CCFES) to cyclic NeuroMuscular Electrical Stimulation (cNMES) with a significant improvement at the BBT in the CCFES group (66).

##### 3.2.2.6. Local vibration stimulus program

Tavernese et al. used segmental muscle vibration over biceps brachii and flexor carpi ulnaris muscles of the paretic side with a significant improvement in the normalized jerk, an indicator of smoothness of the movement, in mean linear velocity, in mean angular velocity at shoulder, in distance to target at the end of movement and movement duration (67). On the other hand, Costantino C. et al. analyzed the short-term effect of local muscle vibration treatment on upper limb obtaining significant results, after 4 weeks, in grip muscle strength, pain and quality of life and decrease of spasticity (68).

#### 3.2.3. Other outcomes

##### 3.2.3.1. Different approaches of trunk exercises regimes

Karthikbabu et al. compared plinth and Swiss ball-based trunk exercise regimes with a standard physiotherapy; after 6 weeks there were significant changes in Trunk Impairment Scale (TIS), BBA, Tinetti scale, gait speed, SIS and community reintegration but they were retained during the 3–12 months follow up (69). In Lee et al. work, there was a comparison of the effects of a Conventional Core Stabilization (CCS) and a Dynamic Neuromuscular Stabilization (DNS) on Anticipatory Postural Adjustment (APA), time, balance performance and fear of falls in chronic stroke patients. After the intervention the APA times changed significantly in the DNS group; the BBS, TIS and Falls Efficacy Scale (FES) scores improved in both groups but with a time stability only in the DNS group (70). Lee et al. investigated the effect on trunk control and dynamic balance ability in the sitting position of a dual motor task training program. After 6 weeks, the experimental group showed significant improvements in trunk control ability and dynamic balance in sitting position (71).

##### 3.2.3.2. Aquatic programs

Park et al. investigated the effects of a Land-based and Aquatic Trunk Exercise program (LATE) in chronic stroke patients; after 4 weeks of intervention there were clinically significant improvements in Korean-TIS, 3-level Postural Assessment Scale for Stroke (PASS 3-L), BBS 3-Level and Modified-BI scores and Functional Reach Test (FRT) distance (72). In Perez-de la Cruz work, the program of Ai Chi aquatic therapy showed that there were significant ameliorations in post treatment pain and resilience; also, they found changes in the SF-36 test except for general health, vitality, and social functions (73). Also, Ku et al. studied the effects of Ai Chi therapy but under a different point of view, focusing on the significant changes seen in Limits of Stability (LOS) test (anteroposterior axis), BBS and FMA (74).

## 4. Discussion

In this systematic review, we conducted the research including randomized controlled trials (RCT) published over the last 10 years with the purpose of identifying all the effective rehabilitation treatments and setting in chronic stroke patients. We also wondered about the possibilities of improvements and the future perspectives for both patients and research. In fact, the mechanism on which recovery can occur even more than 6 months after the acute event has not been fully clarified yet, therefore it cannot be assumed that any therapeutic option will be successful. Insights of this kind could make a significant contribution to the knowledge of this chronic neurological issue and above all to what a patient can probably expect from the course of his disease.

Several authors decided to explore the rehabilitative perspective of robotic technology, obtaining most of the results. In Calabrò et al. and Kooncumchoo et al. studies, two types of gait training machines (Ekso in the first one and I-Walk in the second one) were tested: in both studies there was a significant improvement in 10MWT/speed but only in the first one there were positive changes in gait quality. Even if the I-Walk machine did not show massive advantages compared to conventional therapy, it sufficiently facilitates locomotor function with the setting of task-specific training and an adequate number of repetitions with an appropriate gait pattern (50 step/min). In this way, the patient learns to control new movements with normal biomechanics and to use less energy when performing tasks. In contrast, the repetitions in the conventional PT group were possibly inadequate to control precise movement but the sensory stimulation by the physical therapist provided a more effective patient response and range of motion (28, 29).

Both in Wu et al. and Cho et al. studies, the robotic assistance protocol showed to be superior or not inferior to other protocols. In Wu et al. work, the resistance protocol did not overcome the assistance training protocol in improving endurance, balance, and balance confidence in individuals poststroke. A possible reason is that the motor memory and the acquired cognitive strategies, resulted from the resistance force applied to lower limbs, may be less retained and transferred to overground walking. Certainly, a force perturbation and a controlled assistance load to the paretic leg during treadmill training may be used as an adjuvant tool to improve locomotor functions in poststroke patients, even for subjects of a high functional level (30, 56).

Hung et al. decided to investigate if robot-assisted therapy could bring more improvements either if associated with unilateral or bilateral arm training. In the end. BHT was more effective for improving upper extremity motor function probably because the patients were more likely to use their affected Upper Extremity (UE) with the assistance of the unaffected UE and practiced more in dexterity tasks compared with those in the UHT group. At the same time, UHT is useful in term of enhancing UE motor abilities and physical- related Quality of Life but the results were not maintained at long term. Instead, the robot training group showed higher improvements in functional ambulation (58). On the other hand, Cordò et al. studied an existing protocol using robot-assisted movement combined with local vibration therapy; they wanted to assess if EMG biofeedback or torque biofeedback could improve the recovery of the severe hand impairment. The results were overlapping in both cases (57). Upper Limb function was also used as an outcome in Lin et al. article: the intervention group reached optimal changes in FMA, BI, WMFT and MAS by using a computer-aided bilateral isometric handgrip (59).

A hybrid approach was used by Lee et al. combining robot-assisted therapy with Neuromuscular Electrical Stimulation to implement the functions of the upper limb, in particular of the hand (65). They achieved good results as well as Yang et al. that used NMES on anterior tibialis muscle and medial gastrocnemius muscle (41). Knutson et al. demonstrated the predominant effects of CCFES on cNMES in achieving a finer hand dexterity (66). On the contrary, intervention superiority was not obtained in the study conducted by Bethoux et al. as they used FES on common peroneal nerve com-pared to Ankle Foot Orthosis (42). Beaulieu et al. showed how also RPSM is an effective tool for ameliorations in stroke patients with a paretic tibialis anterior muscle and with spastic plantar flexor muscles (43).

A great technologic resource for recovery in chronic stroke patients is the use of virtual reality of different types (immersive or exergames) in lower limb and balance function. The protocols used by the authors are different, some using Exergame with physical exercise that integrates motion-tracking technology that enables interaction with the game and real-time feedback of user’s performance or immersive VR with deep mental involvement in something action. In the context of virtual reality, and in a technical acceptance of the term, immersion is achieved by removing as many real-world sensations as possible and substituting these with the sensations corresponding to the virtual reality experience, but the patient must have good confidence with the technology and bring back an adequate cognitive reserve, that’s why an adequate MMSE is always required.

Supporting evidence can be found in Kayabinar et al. and in Llorens et al. studies where they both used Virtual Reality combined with robotic technology or with stepping training.

In Kayabinar et al. work, VR was added as support in robot-assisted gait training under the form of an exergame: the experimental group was tasked to walk in a forest environment with many trees, without hitting them and trying to collect the coins that appeared on the screen. The patients determined their direction during the game by transferring weight to their extremities on the device. The protocol lasted 6 weeks, two session/week, for a total of 12 sessions (38).

On the other hand, Llorens et al. associated immersive VR with stepping training to create a protocol of 20 one-hour rehabilitation sessions, 5 days a week for 4 weeks. The experimental group underwent 30 min of conventional therapy and 30 min of training with the virtual rehabilitation system in that order. In this group, the exercises of conventional therapy were administered consecutively in single 5-min repetitions and the training with the virtual rehabilitation system consisted of three 6-min repetitions with one and a half minute breaks between them. The exercise immersed the participants in a 3D virtual environment with their feet represented by two shoes that mimicked their movement in the real world (39).

Also, the combination of immersive VR with MT showed to be an excellent therapeutic choice as confirmed by In et al. where visual illusion was used to help the patient in the recovery of the Lower Limb Function (37).

Instead, as regards the Upper Limb Function (ULF), VR has not proved to be superior compared to home exercises programs as seen in Hernandez et al. (63).

Following the discovery of the mirror neuron system, it has been years since mirror therapy has been used as a fundamental tool for recovery in stroke patients. Therefore, in our research it is not surprising the great number of articles regarding new upgrades of this technique: for example, Arya et al. showed how an activity-based mirror therapy was more effective than the conventional one (32).

Assuming the positive effects of MT, some authors have combined it with other rehabilitation technologies such as afferent electrical stimulation (31) and TDCS (Transcranial Direct Current Stimulation). Minimum collateral effects were found in the second work: a patient dropped out the study for the appearance of a headache (62). The pairing of tDCS with MT may influence restoration of daily function and movement efficiency of the paretic hand in chronic stroke patients. Sequentially applying tDCS prior to MT seems to be advantageous for enhancing daily function and hand movement control and may be considered as a potentially useful strategy in future clinical application. Both these hybrid techniques have reported good results especially regarding muscle strength.

Choi et al. have implemented mirror therapy with a gesture recognition device obtaining significant changes in motor function but also in quality of life and depression (60). A limit of this technique was found by Colomer et al. and relates to the severity of the upper limb impairment: patients with severely impaired UE function, treated with MT, showed some improvements in tactile sensation similarly to the control group in FMA and ability subscales (61).

However, authors agree in suggesting MT even in completely plegic stroke survivors, as it uses visual stimuli for producing a desired response in the affected limb to have effects not just on motor impairments but also on sensations, visuospatial neglect, and pain after stroke. Also, MT is an easy and low-cost method.

Son et al. demonstrated how self-observation training (a specific protocol based on eliciting mirror neuron system) associated with exercise therapy can improve muscle activity of the most important muscles involved in gait (33). Also, a video of treadmill walking action taken at various speeds can be an optimal instrument for the recovery of gait functions as demonstrated by Bang et al. (34). Cho et al. used an innovative program based on motor imagery training where patients were asked to conceive motor patterns associated with gait; there were improvements in many outcomes such as gait abilities and balance (35). The same technique was used by Dickstein et al. but it was performed in a household setting with optimal results (36). Somatosensory training was treated by Alwhaibi et al. and De Diego et al. with the purpose of acquiring some latent function, as actually noticed in the results obtained in the FIM score and in the sensory tests (40, 64). In our research, a technique that obtained excel-lent achievements both in postural sway and in upper limb function was the local vibration stimulus training program (44, 68).

More in detail, Tavernese et al. demonstrated how the segmental muscle vibration can improve, at least in a short-term period, upper limb motor performances of reaching movement (67).

Physical exercise is the basis of rehabilitation treatment after a stroke episode, we therefore tried to define what features it should have to be effective in the chronic stroke patient. For example, high-intensity exercise appears to be effective in gaining gait abilities (stepping amount and rate) and spatiotemporal symmetry as showed by Hornby et al. (47). Its positive influence can also be enhanced by the combination with TENS, placed on the affected lower extremity, as seen in Park et al. work with a reduction of spasticity and an improvement in balance and gait (45).

As a matter of fact, lack of trunk control is usually a critical problem in chronic stroke patient, for this reason plenty of studies are introducing specific new protocols about it.

Karthikbabu et al. tried to demonstrate the efficacy of a plinth and Swiss ball-based trunk exercise regime, but the initial good results were not maintained over the time (12 months follow up) (69). Instead, in the research of Lee et al. the beneficial effects reached with a dual motor task training program were sustained (71). Lee et al. decided to focus on anticipatory postural adjustments (APA) with two different strategies. The one with better results was dynamic neuromuscular stabilization (DNS) with achievements maintained over the time (70). Treadmill Training is another classic fundamental tool that can be used in chronic stroke patient for recovery in gait performance and balance skills as seen in Globas et al. paper where patients improved their walking capacities after a treadmill training (48). In our research we found four studies that tried to ameliorate this technique. Chen I. et al. demonstrated that turning-based treadmill is more effective than the normal one in walking performance (49). This outcome was also studied and improved in Choi et al. work and in Cho et al. work where the treadmill training was associated with a whole-body vibration in the first one and with real-world video recording in the second one (50, 51). Treadmill training was also matched with tilt sensor functional electrical stimulation on common peroneal nerve demonstrating to be a valid instrument in balance, gait and muscle architecture (42).

Aquatic programs in chronic stroke patients seem to be well accepted and performed, presuming an adequate cardiovascular and cognitive condition. Park et al. supplemented a land-based trunk exercise program with an aquatic one in patients with significant improvements in balance, independence, and quality of life (72).

A well specified protocol is Ai Chi which consists of Tai Chi principles applied in water. This form of aquatic exercise involves a total of 19 standardized movement patterns that focus on coordination of body movements with breathing and specific patterns. This practice is safe, standardized, does not require specific equipment, it can be taught in a group setting and allows participants to continue their own. It has been proven to be a valid therapeutic option in improving balance, motor function and in reducing post treatment pain (73, 74).

In our research we found out that other different approaches have been applied to these patients, such as different types of mobilizations (talocrural and self-ankle) as used in An et al. and Park et al. works, which demonstrated how balance and gait parameters can be restored by using them (52, 53).

Instead, Liao et al. established how both lateral wedge soles and visual biofeedback balance training can reinforce balance abilities (55).

Some of the selected studies have shown that chronic stroke patients’ rehabilitation can also be performed in a home environment, with or without the presence of the physical therapist, taking for granted an adequate cognitive condition and ability to understand the instructions: in this way the management of the patient after discharging gets simpler (36, 46, 54, 63, 64).

Moreover, a home-based setting allows greater patient compliance. Home rehabilitation makes it possible to contain the high costs of inpatient rehabilitation programs and improve the continuity of care while patients are transferred to home. Moreover, the possibility of treatment at home allows to reduce the economic costs and makes life easier for the patient and the caregiver and is a valid tool during periods of pandemic such as covid.

After discharge from in-hospital rehabilitation, chronic post-stroke patients should have the opportunity to continue the rehabilitation through structured programs to maintain the benefits acquired during intensive rehabilitation treatment.

## 5. Limitations

The present study represents an overview, through scoping review, of the main rehabilitation techniques commonly used in patients with disabilities secondary to chronic stroke. No comparisons were made between the different neurorehabilitation techniques or the waiting list or with placebo. This comparison will be the subject of future studies.

## 6. Conclusion

Our study provides an overview of the main rehabilitation techniques used in patients with chronic stroke sequelae with different levels of efficacy. For a long time, it was believed that the window of opportunity within which to provide stroke rehabilitation was limited to the first 3–6 months after the acute event. As a result, too often, rehabilitation resources for managing chronic stroke have not been adequate. But when does one reach a phase of stabilized outcomes and is it therefore correct to speak of chronicity? Although the definition of acute phase and chronic phase in terms of recovery is still debated, there are different evidence in support of a rehabilitation continuity in the chronic hemiplegic patient aimed at guaranteeing further results also in the long term. Considering these considerations, our data suggest that a prosecution of the rehabilitation is possible even after the first 6 months, not only as a maintenance treatment but also aimed to acquire or recover some loss functions. Furthermore, we have noticed how a rehabilitation protocol can be ap-plied in an outpatient setting but also at home, and this could increase patient compliance including caregiver support. The reading of this evidence is intricate by methodological factors such as the variety of used scales and outcomes, follow up timing, range and characteristics of the population studied. Nonetheless other studies are needed to establish shared neuro- rehabilitative protocols with respect to the different characteristics of the patients in the chronic post-stroke phase.

## Author contributions

TP, EM, SC, and FS: conceptualization. TP, EM, and SC: methodology. EM and SC: investigation and formal analysis. EM, SC, and MM: data curation. TP, FS, EM, and SC: writing—original draft and preparation. TP, EM, SC, MC, MM, and MP: writing—review and editing. TP, MM, MP, FA, and AB: visualization. TP, MM, and MP: supervision. All authors read and approved the final version of the Manuscript.

## Conflict of interest

The authors declare that the research was conducted in the absence of any commercial or financial relationships that could be construed as a potential conflict of interest.

## Publisher’s note

All claims expressed in this article are solely those of the authors and do not necessarily represent those of their affiliated organizations, or those of the publisher, the editors and the reviewers. Any product that may be evaluated in this article, or claim that may be made by its manufacturer, is not guaranteed or endorsed by the publisher.
